# Supplementary material for: A general method for determining secondary active transporter substrate stoichiometry
Source: eLife. 2017 Jan 25;6:e21016. doi: 10.7554/eLife.21016 (PMC5305207; doi:10.7554/eLife.21016)
Supplement: Supplementary file 1. — Constituent concentrations of internal and external buffers used in each experiment. ICD stands for internal concentration determination. Columns are color-coded to match the experimental schematics presented in each figure. DOI: http://dx.doi.org/10.7554/eLife.21016.009 [file elife-21016-supp1.docx]

**Supplementary Table 1**

**
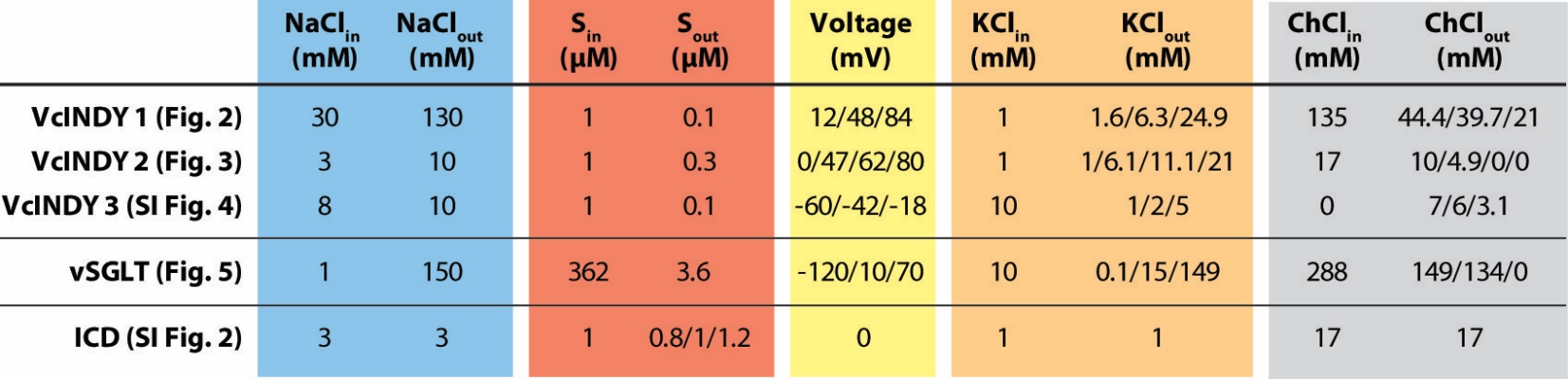
**

**Supplementary Table 1. Exact buffer conditions for all experiments presented.** Constituent concentrations of internal and external buffers used in each experiment. ICD stands for internal concentration determination. Columns are color-coded to match the experimental schematics presented in each figure.
